# Influence of Food Matrices and the Population of Interfering Microorganisms on the Determination of *Listeria monocytogenes* by Conventional Methods and VIDAS

**DOI:** 10.3390/foods10123021

**Published:** 2021-12-06

**Authors:** Maribel Estévez, Fernando García-Viejo, Mª Carmen López-Mendoza, Rafael Jordano, Luis M. Medina

**Affiliations:** 1 Department of Food Science and Technology, Campus of Rabanales, University of Córdoba, Ctra. NIV, km 396a, E-14071 Córdoba, Spain; maribelred@hotmail.com (M.E.); rjordano@uco.es (R.J.); 2 Public Health Laboratory, Delegación Provincial de Salud, E-14071 Córdoba, Spain; mical@uco.es; 3 Department of Food Science and Technology, University CEU-Cardenal Herrera, Edificio Seminario s/n., Moncada, E-46113 Valencia, Spain; clopez@uchceu.es

**Keywords:** *Listeria*, food matrices, conventional methods, interfering microorganisms, VIDAS

## Abstract

In this study, the possible influence of the food matrix and the interfering population of microorganisms on the detection and count of *Listeria monocytogenes* in three common foods of the Spanish diet (Spanish omelette, fresh cheese and vegetable salad) was determined. Four groups were assayed: one control, two groups with interfering microorganisms (*Salmonella* Enteritidis, *Staphylococcus aureus* and *Proteus mirabilis*) with different levels of *L. monocytogenes* and a final group only contaminated with *L. monocytogenes*. The samples were analyzed with the normalized method (UNE-EN ISO 11290:2018) and with an alternative technique (VIDAS). The results show that the presence of interfering microorganisms did not seem to interfere with the determination of *L. monocytogenes*. Furthermore, the type of food did not seem to influence the determination of *L. monocytogenes*, but the culture media used showed differences. In fact, regardless of the type of food, the ALOA medium showed higher sensitivity than the other media, with higher recovery in 100% of samples (only for the Spanish omelette in Group B was the result the same as that for PALCAM, −8.11 log cfu/g). The results obtained using the VIDAS were not influenced by any of the factors or conditions used and show 100% efficiency.

## 1. Introduction

*L. monocytogenes* is a microorganism of high relevance in analyses of food safety criteria checks [1]. Since 2000, an increase in the number of listeriosis cases has been observed in different European countries, but the reasons for this phenomenon remain unclear [2,3]. The detection of *L. monocytogenes* in food has very important sanitary [4] and economic consequences, leading to withdrawal of products [3] as well as reputational costs.

For the food industry, the main disadvantage of conventional methods for the detection of *L. monocytogenes* [5,6] is the time required to obtain the final results, and that, generally, they are often not sensitive enough for foods contaminated with *L. monocytogenes* and/or *Listeria* spp. [7]. Between the alternative methods available, the VIDAS (Vitek Immuno Diagnostic Assay System), based on ELFA (enzyme linked fluorescent assay), provides a significant reduction in the time needed for the detection of pathogens in foods [8].

Various works recently compared some of these methods. Bustamante et al. (2020) [9] isolated *L. monocytogenes* according to the ISO 11290-1:2017 standard, detected with the VIDAS and identified by PCR (polymerase chain reaction), in ready-to-eat artisanal Chilean foods. Additionally, Ríos-Castillo et al. (2020) [10] studied the capacity of real-time PCR (RT-PCR), the VIDAS and conventional methods for detecting *L. monocytogenes* biofilm cells.

In previous works, the sensitivity of different alternative methods for the isolation and identification of pathogens in poultry [11] and the incidence of *L. monocytogenes* in refrigerated and frozen chicken parts by the VIDAS [12] were also evaluated.

In this work, we attempted to assess the possible influence of different food matrices (Spanish omelette, fresh cheese and vegetable salad) and the population of interfering microorganisms (*Salmonella* Enteritidis, *Staphylococcus aureus* and *Proteus mirabilis*) on the determination of *L. monocytogenes* by conventional methods [5,6] and the VIDAS.

## 2. Materials and Methods

### 2.1. Sampling and Description of Samples

The food matrices analyzed were: precooked Spanish omelette (potato, 51%; pasteurized egg, 24%; onion, 13%; olive oil, 2%; salt; emulgent, lecitin of soybean; stabilizers, E-407, E-415 and E-410; natural aroma; pH: 7.45; a_w_: 0.96); fresh cheese (pasteurized cow’s milk, salt and rennet; minimum of 28% of dry matter (DM) and 45% fat in dry matter (FDM); pH: 6.10; a_w_: 0.99); and vegetable salad packaged under a modified atmosphere (MAP) (Chinese cabbage, red cabbage, carrots and cherry tomato; pH: 6.30; a_w_: 0.98). Once the different foods were available (omelette, cheese and salad), four groups were prepared (A, B, C and D) with two levels of contamination with *Listeria*. From each group, 4 samples were obtained (48 samples).

Groups of each food were defined as: Group A (control); Group B: foods inoculated with a low concentration of *L. monocytogenes* and the interfering microorganisms (*S.* Enteritidis, *S. aureus* and *P. mirabilis*); Group C: foods inoculated with a higher concentration of *L. monocytogenes* and the interfering microorganisms; Group D: foods inoculated with a low concentration of *L. monocytogenes*.

### 2.2. Experimental Contamination of Samples

Cultures of *L. monocytogenes* and different interfering microorganisms were prepared. The cultures were prepared from cryogenized strains belonging to the Spanish Culture Type Collection (CECT): *L. monocytogenes* CECT 5873; *Salmonella enterica* ssp. *enterica* serovar Enteritidis CECT 4300; *Staphylococcus aureus* ssp. *aureus* CECT 976; and *Proteus mirabilis* CECT 5350. Twenty-five grams of the food was contaminated with or without interfering microorganisms and *L. monocytogenes*, as necessary (groups A, B, C and D). The two levels of *Listeria* contamination were 10^2^ cfu/g for the lower level and 10^3^ cfu/g for the higher level of contamination. The level of contamination for interferent microorganisms was 10^3^ cfu/g, and the adherence time used was 4 h.

### 2.3. Count of Listeria Monocytogenes by Conventional and ISO Methods

Different dilutions of each sample were prepared in order to determine *Listeria monocytogenes* counts by a conventional method [5], using PALCAM Medium, and the current normalized method (UNE-EN ISO 11290-2:2018) [6]. The counts were expressed in log cfu/g.

### 2.4. Detection of Listeria monocytogenes by VIDAS

Twenty-five grams of sample was placed in 225 mL of Fraser broth (bioMérieux), homogenized in the stomacher for 2 min and incubated at 30 °C for 24 h. One-milliliter aliquots of these primary enrichments were transferred to 1 mL of secondary enrichment Fraser broth (bioMérieux) and incubated at 30 °C for 24 h. Finally, 0.5 mL was inoculated into the sample wells of the strips provided by VIDAS-LMO II (for the detection of *L. monocytogenes*). An RFV of ≥0.05 for one sample was considered as a presumptive-positive result. The results were obtained after 70 min and were expressed as presence or absence of *L. monocytogenes*.

### 2.5. Statistical Analyses

Statistical analysis was carried out using the SPSS 15.0 Software package (IBM Company, Armonk, NY, USA). The efficiency of the tested measures was determined by comparing results according to the different parameters studied. For this purpose, a non-parametric chi-square (Χ^2^) test with a level of significance of 5% (*p* < 0.05) was performed.

## 3. Results

The results obtained corresponding to the four groups (A, B, C and D) of each food are summarized in Table 1 and Table 2.

Both in samples inoculated only with *Listeria* and in those contaminated with other microorganisms (*Salmonella*, *Staphylococcus* and *Proteus*), the results show that the presence of interfering microorganisms did not seem to interfere with the determination of *L. monocytogenes*. In particular, the VIDAS showed 100% efficiency.

Regardless of the type of food, the ALOA medium showed higher sensitivity than the other media, with higher recovery in 100% of samples (only for the Spanish omelette in Group B was the result the same as that for PALCAM, −8.11 log cfu/g). On the other hand, the Oxford medium was less effective. The differences between the recovery in the different media were significant (<0.05).

Regarding the different foods, the salad showed higher counts of *Listeria*, although no significant differences were demonstrated. Thus, in the PALCAM and ALOA media, the highest count of *Listeria* was clearly obtained in salad samples, whereas the counts in the fresh cheese and omelette were relatively similar, although the ALOA medium seemed to work better for the cheese, and PALCAM for the omelette. However, in the cheese, some interferences and erroneous staining were observed with the ALOA medium. For the Oxford medium, the highest counts were reported in the fresh cheese. With application of the VIDAS, no incidence was determined.

On comparing results with and without interfering microorganisms, we could not establish statistical differences, as it can be expected after observing data.

## 4. Discussion

Golden et al. (1987) [13] used a direct plate culture, with a high level of sensitivity, with no previous enrichment to recover *L. monocytogenes* from milk and ice cream. On the contrary, when assessing foods (raw pumpkin and Brie cheese) with high inoculates of interfering microorganisms, direct plate *L. monocytogenes* recovery was not so satisfactory. In Blysick-McKennal et al.’s (1994) [14] opinion, the methodology for the enumeration of *Listeria* spp. in food is complex in cases of low rates of those bacteria and high concentrations of competitive biota. In our case, the presence of interfering microorganisms did not seem to influence the determination or counts of *L. monocytogenes*, either by the traditional method or by the VIDAS. However, the influence of interfering microorganisms at the higher or lower sensitivities of conventional media is open to consideration for further studies.

Regarding the possible influence of the medium used, ISO [5] considers PALCAM as a suitable method to recover *L. monocytogenes*. Aguado et al. (1996) [15] recovered a larger number of *L. monocytogenes* in frozen vegetables and in cooked meat products with L-PALCAM. In the present work, in the determination carried out using the traditional methodology, the influence of the type of food according to the medium used was not significant, although for PALCAM and ALOA, the lowest counts were obtained in the fresh cheese and omelette, and the highest ones in the salad, whereas for the Oxford medium, the highest count was obtained in the fresh cheese.

The ALOA medium was recently included in the ISO 11290 method, after demonstrating its specificity for the confirmation of *L. monocytogenes* [7]; *Listeria ivanovii* (the only species which could return false positives if only morphological evaluation of the colony is considered) was not identified using this medium. The detection limit ranged from 1 to 2 log cfu/g using Fraser enrichment broth. The sensitivity of the method permitted the detection of low contamination levels (<1 log cfu/25 g). Artault et al. (2000) [7] demonstrated the precision of this medium (100% coincidence between the reference method and ALOA). Similarly, Vlaemynck et al. (2000) [16], using the ALOA medium, detected 4.3% more positives than with the ISO method in dairy product and meat samples. They found 13.9% of false negatives, while with the traditional methods of PALCAM/Oxford, they detected 38.9% of false negatives. For these authors, ALOA clearly shows a higher sensitivity than PALCAM and Oxford when the samples contain *L. monocytogenes* and *Listeria innnocua*. In our case, the counts of *L. monocytogenes* in ALOA were higher than those obtained using the other media, the Oxford medium being the least effective. Campos Mata et al. (2016) [17] showed that the conventional method provided a better recovery of *L. innocua* throughout artisanal Minas cheese ripening in artificially contaminated samples than immunoanalytical methods, which is probably due to the interference of the intrinsic characteristics of the artisanal cheeses.

According to Reiter et al. (2010) [11], to determinate *Listeria*, the plate count method proved less sensitive than the VIDAS, the VIP (Visual Immunoprecipitate Assay) system and the Reveal system, which yielded similar results. For these authors, in a general way, the VIDAS appeared to be an effective alternative to traditional methods. Indeed, our results confirm that the VIDAS was valid as a routine procedure. Additionally, in all the assumptions made, the VIDAS method showed a correlation of 100% with the results of the presence or absence of *L. monocytogenes*. Numerous examples have already been published describing the use of molecular methods to subtype and source *L. monocytogenes* on farms, in plants and/or in foods, or for comparison [18]. Previously, Uyttendaele et al. (1995) [19] proved a 100% correlation in most of the cases studied by comparing three methods (NASBA, nucleic acid sequence-based amplification; an ELISA-based method; and a modified cultural method proposed by the US Food and Drug Administration (FDA)) in a variety of artificially inoculated foods (chicken breast meat, minced meat, shrimp, raw milk, soft cheese, dry sausage, mushroom and radish).

## 5. Conclusions

The previous experience and our results in this work show a general coincidence between the conventional methodology and other methods such as the VIDAS. In our case, it was found that there were no notable differences between the samples inoculated with *Listeria* and those which were also contaminated with interfering microorganisms; however, the influence of interfering microorganisms at the higher or lower sensitivities of conventional media could be studied further. Furthermore, the type of food matrices seemed to have no influence on the determination of *L. monocytogenes*. The results obtained through VIDAS application were not influenced by any of the factors or conditions used and show high efficiency in the detection of *Listeria*, with 100% of coincidence between detection and experimental contamination.

## Figures and Tables

**Table 1:** Results ^a^ of the determination of *Listeria monocytogenes* in different foods by traditional methods and the VIDAS (*n* = 4 for each group of foods).

Media/Dilution	Group A			Group B			Group C			Group D		
	SO	FC	VS	SO	FC	VS	SO	FC	VS	SO	FC	VS
PALCAM	<10	<10	<10	6.30	6.30	6.95	8.11	7.95	8.53	6.30	6.26	6.95
Oxford	<10	<10	<10	5.08	5.08	5.08	6.71	6.79	6.69	5.18	5.34	5.18
ALOA	<10	<10	<10	7.40	7.48	8.38	8.11	8.27	8.65	7.30	7.48	8.45

^a^ log cfu/g; Group A: control; Group B: inoculated with *L. monocytogenes* (2 log cfu/g), *S.* Enteritidis, *S. aureus* and *P. mirabilis* (3 log cfu/g); Group C: inoculated with *L. monocytogenes* (3 log cfu/g), *S.* Enteritidis, *S. aureus* and *P. mirabilis* (3 log cfu/g); Group D: inoculated with *L. monocytogenes* (2 log cfu/g); SO: Spanish omelette; FC: fresh cheese; VS: vegetable salad.

**Table 2:** Results of the determination of *Listeria monocytogenes* by the VIDAS (*n* = 4 for each group of foods).

Media/Dilution	Group A			Group B			Group C			Group D		
	SO	FC	VS	SO	FC	VS	SO	FC	VS	SO	FC	VS
10^−1^	−	−	−	+	+	+	+	+	+	+	+	+
10^−2^	−	−	−	+	+	+	+	+	+	+	+	+
10^−3^	−	−	−	+	+	+	+	+	+	+	+	+
10^−4^	−	−	−	+	+	+	+	+	+	+	+	+
10^−5^	−	−	−	+	+	+	+	+	+	+	+	+
10^−6^	−	−	−	+	+	+	+	+	+	+	+	+
10^−7^	−	−	−	+	+	+	+	+	+	+	+	+

Group A: control; Group B: inoculated with *L. monocytogenes* (2 log cfu/g), *S.* Enteritidis, *S. aureus* and *P. mirabilis* (3 log cfu/g); Group C: inoculated with *L. monocytogenes* (3 log cfu/g), *S.* Enteritidis, *S. aureus* and *P. mirabilis* (3 log cfu/g); Group D: inoculated with *L. monocytogenes* (2 log cfu/g); SO: Spanish omelette; FC: fresh cheese; VS: vegetable salad; +: presence; −: absence.

## References

[1] Anonymous (2007). Commission regulation (EC) No 1441/2007 of 5 December 2007. Amending regulation (EC) No 2073/2005 on microbiological criteria for foodstuffs. Off. J. Eur. Union.

[2] Anonymous (2015). European Food Safety Authority and European Centre for Disease Prevention and Control, 2015. The European Union summary report on trends and sources of zoonoses, zoonotic agents and food-borne outbreaks in 2014. EFSA J..

[3] Gnanou Besse N.G., Lombard B., Guillier L., François D., Romero K., Pierru S., Bouhier L., Rollier P. (2019). Validation of standard method EN ISO 11290-Part 1–Detection of *Listeria monocytogenes* in food. Int. J. Food Microbiol..

[4] Magalhaes R., Mena C., Ferreira V., Silva J., Almeida G., Gibbs P., Teixeira P., Motarjemi Y. (2014). Bacteria: *Listeria monocytogenes*. Reference Module in Food Science. Encyclopedia of Food Safety.

[5] ISO 11290-1 & 2 (1998). Horizontal method for the detection and enumeration of *Listeria monocytogenes*. Part 1: Detection method. Part 2: Enumeration method. Int. J. Food Microbiol..

[6] Anonymous (2017). EN ISO 11290-1, Microbiology of the Food Chain—Horizontal Method for the Detection and Enumeration of Listeria Monocytogenes and of Listeria Spp.—Part 1: Detection Method.

[7] Artault S., Bind J.L., Delaval J., Dureuil Y., Gaillard N. (2000). AFNOR Validation of the Method for the Detection of Listeria Monocytogenes in Foodstuffs.

[8] Priego R., Medina L.M., Jordano R. (2009). Comparison between the Vitek Immunodiagnostic Assay System and PCR for the detection of pathogenic microorganisms in an experimental dry sausage durin its curing process. J. Food Prot..

[9] Bustamante F., Maury-Sintjago E., Cerda Leal F., Acuña S., Aguirre J., Troncoso M., Figueroa G., Parra-Flores J. (2020). Presence of *Listeria monocytogenes* in ready-to-eat artisanal Chilean foods. Microorganisms.

[10] Ríos-Castillo A.G.G., Ripolles-Avila C., Rodríguez-Jerez J.J. (2020). Detection of Salmonella Typhimurium and *Listeria monocytogenes* biofilms cells exposed to different drying and pre-enrichment times using conventional and rapid methods. Int. J. Food Microbiol..

[11] Reiter M.G.R., López M.C., Jordano R., Medina L.M. (2010). Comparative study of alternative method for safety control in poultry slaugherhouses. Food Anal. Methods.

[12] Reiter M.G.R., Volkmann H., Imianovsky U., López M.C., Medina L.M., Jordano R. (2011). Listeria monocyogenes in refrigerated and frozen chicken parts. Acta Aliment..

[13] Golden D.A., Beuchat L.R., Brackett R.E. Direct planting technique for enumeration of *Listeria monocytogenes* in foods. Proceedings of the Workshop on Listeria Methodology, 101st AOAC, Annual International Meeting.

[14] Blysick-McKennal D.N., Schaffner D.W. (1994). Prediction of most probable number of *L. monocytogenes* using a generalized linear model and a modified FDA Listeria isolation method. J. Food Prot..

[15] Aguado V., Vitas A., García-Jalón I. Estudio comparativo de tres métodos de enriquecimiento para la recuperación y aislamiento de *L. monocytogenes* en alimentos (comparative study on three metods of enrichment for the recuperation and isolation of *L. monocytogenes* in fodds). Proceedings of the X Congreso de Microbiología de los Alimentos.

[16] Vlaemynck G., Lafarge V., Scotter S. (2000). Improvement of the detection of *Listeria monocytogenes* by the application of ALOA, a diagnostic, chromogenic isolation medium. J. Appl. Microbiol..

[17] Campos Mata G.M.S., Martins E., Gonçalves Machado S., Soares Pinto M., Fernandes de Carvalho A., Danta Vanetti M.C. (2016). Performance of two alternative methods for Listeria detection throughout Serro Minas cheese ripening. Braz. J. Microbiol..

[18] Porto-Fett A.C.S., Call J.E., Muriana P.M., Freier J.A., Luchansky J.B., Juneja V.K., Sofos J.N. (2010). *Listeria* *monocytogenes*. Pathogens and Toxins in Foods.

[19] Uyttendaele M.R., Schukkink R., Van Gemen B., Debevere J.M. (1995). Development of NASBA, a nucleic acid amplification system, for identification of *Listeria monocytogenes* and comparison to ELISA and modified FDA method. Int. J. Food Microbiol..

